# Development of a Wearable Sensor Platform for Fall Risk Assessment and Fall Detection

**DOI:** 10.3390/s26185867

**Published:** 2026-09-16

**Authors:** Antonella Imperato, Michele Caporaso, Valentina De Pascalis, Giuseppe Di Gironimo, Antonio Lanzotti, Stanislao Grazioso, Angela Palomba, Teodorico Caporaso

**Affiliations:** 1 ETA Bioengineering S.R.L., Corso Nicolangelo Protopisani 70, 80146 Naples, Italy; antonella.imperato@etabioengineering.com (A.I.); michele.caporaso@etabioengineering.com (M.C.); valentina.depascalis@etabioengineering.com (V.D.P.); 2 Department of Industrial Engineering—Fraunhofer JL IDEAS, University of Naples Federico II, Piazzale Tecchio 80, 80125 Naples, Italy; giuseppe.digironimo@unina.it (G.D.G.); antonio.lanzotti@unina.it (A.L.); stanislao.grazioso@unina.it (S.G.); 3 Department of Public Health, University of Naples Federico II, Via Sergio Pansini 5, 80131 Naples, Italy; angela.palomba@unina.it

**Keywords:** fall prevention, fall detection, inertial measurement units, surface electromyography, smart clothing, machine learning, deep learning

## Abstract

**Objective:** Falls among the elderly constitute a major global public health issue. Research has therefore focused on two complementary areas: fall risk prevention and fall detection. Existing solutions mainly rely on body-attached sensors confined to laboratory settings, whereas recent research has shifted toward wearable e-textiles with non-invasive, long-term-wear sensors. In this context, the study presents a wearable sensor platform to both predict fall risk and detect falls, based on sensorized clothing integrating inertial measurement units and surface electromyography sensors. **Methods:** Fall risk was estimated from gait parameters extracted during a 10 m walking test as the probability of belonging to a faller (vs. non-faller) group, using a logistic regression model trained on the G-STRIDE dataset, complemented by neuromuscular parameters extracted from sEMG and associated with fall risk. Fall detection, focused on improving pre-impact identification, was framed as a binary classification between activities of daily living and falls, using a reduced Spatio-Temporal Attention Network trained on the FallTL dataset and refined with our own platform’s data. **Results:** The obtained results were: fall-risk assessment, Area Under the Curve = 77.8%, Accuracy = 68.7%; fall detection, Accuracy = 96.7%, F1 Score = 77.3%, lead time = 390 ms. **Conclusions:** As a single-subject proof of concept, these results support the feasibility of a unified platform integrating objective fall-risk screening and fall event identification, with a predicted time before impact suitable for protective systems intervention; validation on a larger, representative cohort is required before any clinical claim can be made.

## 1. Introduction

Falls in the elderly population represent a major global public health issue. According to the World Health Organization (WHO), falls constitute the second leading cause of unintentional injury-related deaths worldwide, resulting in an estimated 684,000 annual fatalities globally [1]. In the United States, recent data indicates that over 25% of adults of age ≥ 65 years experience falls annually, with approximately 37% of these incidents resulting in injuries requiring medical treatment [2]. This high prevalence places a substantial burden on national healthcare systems; for instance, the financial expenditure for non-fatal falls alone reached $80 billion in 2020 [3], and these numbers are going to increase [4]. Beyond the physical trauma, falls are also associated with their psychological effects: the fear of falling can significantly impair autonomy and overall quality of life by leading an individual in a spiraling loop, wherein self-imposed activity restriction results in physical deconditioning, social isolation, and a consequent increased risk of future falls [5].

The increased incidence of falls in older adults has also been linked to neuropsychological changes associated with brain aging. Structural changes, including shrinkage of the frontal and prefrontal lobes and reductions in cortical gray and white matter, are thought to underlie declines in global cognition, attention, executive function, and processing speed, each of which has been prospectively associated with a higher incidence of falls [6]. As a consequence, older adults are thought to increasingly rely on conscious, attention-demanding motor control for tasks such as walking that are normally automatic in younger adults, reducing the cognitive resources available to respond to unexpected balance loss. These mechanisms are further compromised in specific pathological conditions: mild cognitive impairment, a transitional stage toward Alzheimer’s disease, and late-life depression have both been associated with reduced frontal gray and white matter integrity and with an increased risk of falling.

Over the last decade significant effort has been dedicated to fall prevention. In particular, the literature has branched into two primary branches: fall prevention on one side, and real-time fall detection on the other.

Fall prevention begins with an early risk screening and stratification phase before any ambulatory hazard occurs. Historically, it has relied on standardized clinical tests of gait, mobility, and balance, including the 10 m Walk Test (10 mWT), the Timed Up and Go (TUG) test, and the Berg Balance Scale (BBS), often complemented by self-reported fall history questionnaires [7,8,9,10,11]. Over the years, contemporary literature has documented the integration of wearable sensors into standardized protocols to automate and objectify this screening process [12,13,14,15]. Above all, sensors such as Inertial Measurement Units (IMU) enable the automated segmentation of activities of daily living (ADLs) into meaningful sub-phases that can be analyzed individually [15,16,17,18]. In the context of fall prevention, the segmentation of ADLs, such as walking, is particularly relevant as it allows the derivation of clinically meaningful parameters, including gait speed, stride and step duration, cadence and double-support time, that provide valuable insights for assessing fall risk and characterizing frailty-related mobility impairments in older adults [19,20,21,22]. IMUs-derived parameters, however, ignore the underlying causes of impaired mobility: neuromuscular alterations, for example, may affect lower-limb coordination and reduce the ability to generate rapid corrective responses during unexpected perturbations. For this reason, contemporary frameworks incorporate surface electromyography (sEMG), which enables the objective characterization of these alterations through the extraction of relevant muscle information during ADLs. In particular, sEMG-derived metrics, including co-contraction indexes (CCI) obtained from the root mean square (RMS) activation of agonist and antagonist muscles, and frequency-domain features capturing fatigue-related changes in muscle activity, provide valuable insights into neuromuscular factors associated with an increased risk of falls [23,24,25].

Within the broader fall-related literature, fall detection constitutes the second major research branch and, in turn, has developed around two main paradigms: fall detection (FD) and pre-impact fall detection (PIFD). While early approaches mainly focused on FD, triggering alerts after the fall occurred [26], recent research has shifted toward proactive strategies aimed at identifying near-fall events before ground impact occurs [27,28]. This requires real-time processing of large amounts of data, as distinguishing early balance-loss events from normal movements relies on subtle alterations in spatio-temporal patterns. Consequently, fixed-threshold approaches are insufficient for this purpose [29]. Addressing this challenge has therefore motivated the development of attention-based deep learning architectures that selectively weight the most informative time instants and signal channels [30].

Effective fall detection, however, requires continuous monitoring in a real-world domestic environment. Therefore, wearable solutions must be minimally intrusive and comfortable for long-term everyday use. In this context, electronic textiles (e-textiles) emerge as particularly suitable solutions. E-textiles are fabrics in which sensing and electronics are directly embedded into the garment by conductive yarns, textile (dry) electrodes, and integrated modules, so that relevant signals can be acquired during everyday activities while preserving the comfort and wearability of ordinary clothing [31,32].

For fall-risk assessment, the use of e-textiles remains quite limited. To the best of our knowledge, no garment is specifically designed to stratify fall-risk against validated clinical scales or normative gait profiles. Existing e-textiles targeting lower limbs primarily focus on the neuromuscular characterization of ADLs; for example, a wearable platform combining a pressure-sensing insole with an sEMG sleeve has been used to autonomously identify muscles’ activation patterns [33]. The landscape is different for fall detection, where several e-textile solutions have been proposed. Smart socks embedding reusable sEMG electrodes over the tibialis anterior and gastrocnemius muscles have been developed for pre-impact fall detection [34,35]. Similarly, sEMG-integrated shorts and calf sleeves instrumenting the proximal and distal leg muscles have been used to predict freezing-of-gait episodes in Parkinson’s disease, a major contributor to falls in advanced stages [36]. A purely inertial approach has also been explored through a smart sock embedding a motion-sensing yarn, which enables the recognition of activities of daily living, falls, and near-fall events [37].

As shown in the studies discussed above, although fall-risk assessment and fall detection address complementary objectives, they are investigated as distinct research areas, with limited integration, particularly within a single wearable platform based on e-textile technology. To bridge this dual gap, this work introduces a novel branch of the MyoETA system, a sensorized shorts garment with a dedicated tool specifically engineered for fall detection and prevention. The proposed framework seamlessly integrates intelligent screening and active pre-impact prevention within a single wearable device, operating in two successive stages:

- **Stage I**. The device serves as an intelligent clinical screening tool, starting from data acquired during a walking test to stratify an individual’s risk of falling and expressed as the probability of having a walking pattern belonging to a faller (vs. non-faller) group rather than a probability of future falls. To this end, it combines lumbar inertial sensing and textile sEMG technology to acquire complementary kinematic and neuromuscular information.
- **Stage II**. The same device can be used for continuous daily monitoring, acting as a predictive system, particularly for individuals identified as high-falling risk. In this stage, a STA-Net-based algorithm processes the acquired signals in real-time to detect early signs of instability and predict falls during activities of daily living.

The objectives of this work are: (i) to develop and validate, as a first proof-of-concept, a fall-risk screening tool that combines lumbar inertial sensing and textile sEMG technology within a single e-textile garment; (ii) to implement and evaluate a real-time, pre-impact fall-detection algorithm operating on the same platform, so that fall-risk stratification and continuous fall monitoring are integrated within a single wearable device.

## 2. System Architecture

**Figure 1:**
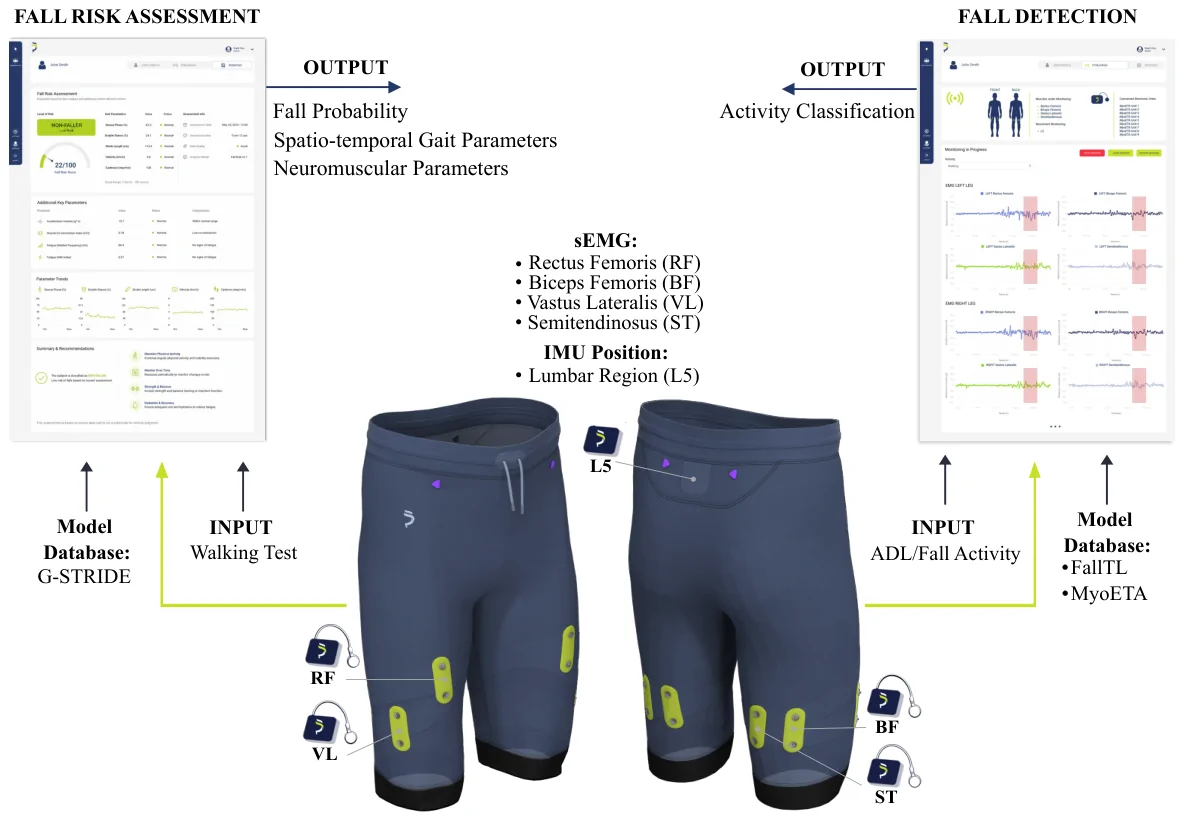
MyoETA System Architecture. The sensorized shorts integrate sEMG sensors positioned bilaterally over four thigh muscles and an IMU located at the L5 vertebral level. Sensor placement is identified by pelvic anatomical landmarks (purple triangles) placed in correspondence with the anterior superior iliac spines (ASIS) and posterior superior iliac spines (PSIS). The figure also shows the two developed pipelines: the fall-risk assessment and the fall-detection modules, together with their respective inputs and outputs.

The used wearable system (MyoETA, ETA Bioengineering S.R.L., Naples, Italy) is made of sensorized shorts designed to integrate sEMG and IMU sensors with a dedicated software for data collection, processing and analysis. The system allows the simultaneous acquisition of muscle activations and kinematics data during ADLs, useful for risk of fall evaluation. In Figure 1 the system architecture is shown.

The base textile is made of a composite material designed to provide adequate adhesion and stable contact with the body during dynamic tasks. Bipolar dry electrode pairs are integrated into the garment and positioned according to the SENIAM guidelines [38,39] above four muscles on both thighs, respectively rectus femoris (RF), vastus lateralis (VL), biceps femoris (BF), and semitendinosus (ST). The electrodes feature a bio-compatible silicone interface that provides reduction in skin-electrode impedance. Consistent with the general mechanism reported for compliant dry electrodes [40], the silicone surface conforms closely to the skin’s microscopic folds, pores, and hair follicles, increasing the effective contact area between the electrode and the skin and thereby reducing the resistive component of the skin-electrode contact impedance. This mechanism is further complemented by the compressive action of the garment itself: mechanical pressure applied to dry electrodes has been shown to produce a comparably large decrease in skin-electrode impedance, similarly attributable to an increase in the effective electrode contact area [41].

To facilitate the correct and repeatable positioning of the garment, heat-sealed visual markers (purple triangles, shown in the figure) have been integrated onto its surface and placed at the following anatomical landmarks: Anterior Superior Iliac Spines (ASIS) and Apollo’s (men) or Venus’s (women) dimples, located at the Posterior Superior Iliac Spines (PSIS). The sensing units are external modules electrically connected to the garment via snap buttons located at the electrode sites. These units are responsible for data acquisition and host the sEMG and IMU sensing hardware. Each unit shares the same hardware and software architecture, allowing flexible configuration for sEMG-only, IMU-only, or combined acquisition. When a single unit is configured for combined sEMG and IMU acquisition, the two channels are sampled by the same on-board clock, with the sEMG sampling frequency set as an integer multiple of the IMU sampling frequency; the firmware acquires one IMU sample every N sEMG acquisition cycles (N being this integer ratio), ensuring deterministic, sample-accurate alignment between the two channels within that unit. For MyoETA the eight sensing units above the muscles are configured as sEMG (sample frequency 2000 Hz); a single sensing unit is configured as an IMU (BMI088, Bosch Sensortec, Reutlingen, Germany; sample frequency 200 Hz) that integrates a tri-axial accelerometer (±3 g) and a tri-axial gyroscope (±2000°/s). This unit is placed at the level of the fifth lumbar vertebra (L5), a location often adopted in the fall-risk assessment for its proximity to the body’s center of mass [42].

A dedicated web-based host platform provides functionalities for sensors connection and configuration, along with real-time data visualization, processing and storage. Data transmission between the sensing units and the host platform is managed via a Bluetooth Low Energy (BLE) protocol, connecting each unit to a dedicated service running locally on the clinician’s PC. This local service issues acquisition commands to the sensing units, requests a real-time preview of the incoming data—streamed at a reduced frequency relative to the original acquisition rate, for visualization purposes only—and, upon a dedicated end-of-acquisition command, downloads the complete, full-resolution dataset recorded during the test. The downloaded data are then transmitted to the web-based platform, where they are stored, processed, and associated with the corresponding patient record for remote analysis.

In the MyoETA configuration, the eight sEMG and the single IMU channels are hosted on physically separate, independently clocked sensing units. To mitigate clock drift across them, an internal time-verification protocol is applied, in which the acquisition controller periodically queries each unit’s local clock (query interval: 1 s).

Building on this common dashboard, two dedicated software tools have been developed, respectively:

- The Fall Risk Assessment tool exploits the full sensing configuration by combining the lumbar IMU with the eight sEMG channels acquired from both thigh muscles. The user is asked to perform a walking test, at the end of which two outputs are provided: the probability of belonging to the faller (vs. non-faller) group, P(faller), based on a logistic regression model obtained from an open-source dataset reported in [43], and a set of additional neuromuscular indices that are well documented in the literature for their association with fall risk.
- The Fall Detection tool solely relies on the lumbar IMU. During ADLs execution, it performs real-time classification between ADLs and PIFD, using a model trained on preliminary data from an open-source database [44] integrated with additional data. The web platform displays the activity classification in real time.

In the next two sections, the methodology used to realize Fall Risk Assessment and Fall Detection are presented.

## 3. Fall Risk Assessment

**Figure 2:**
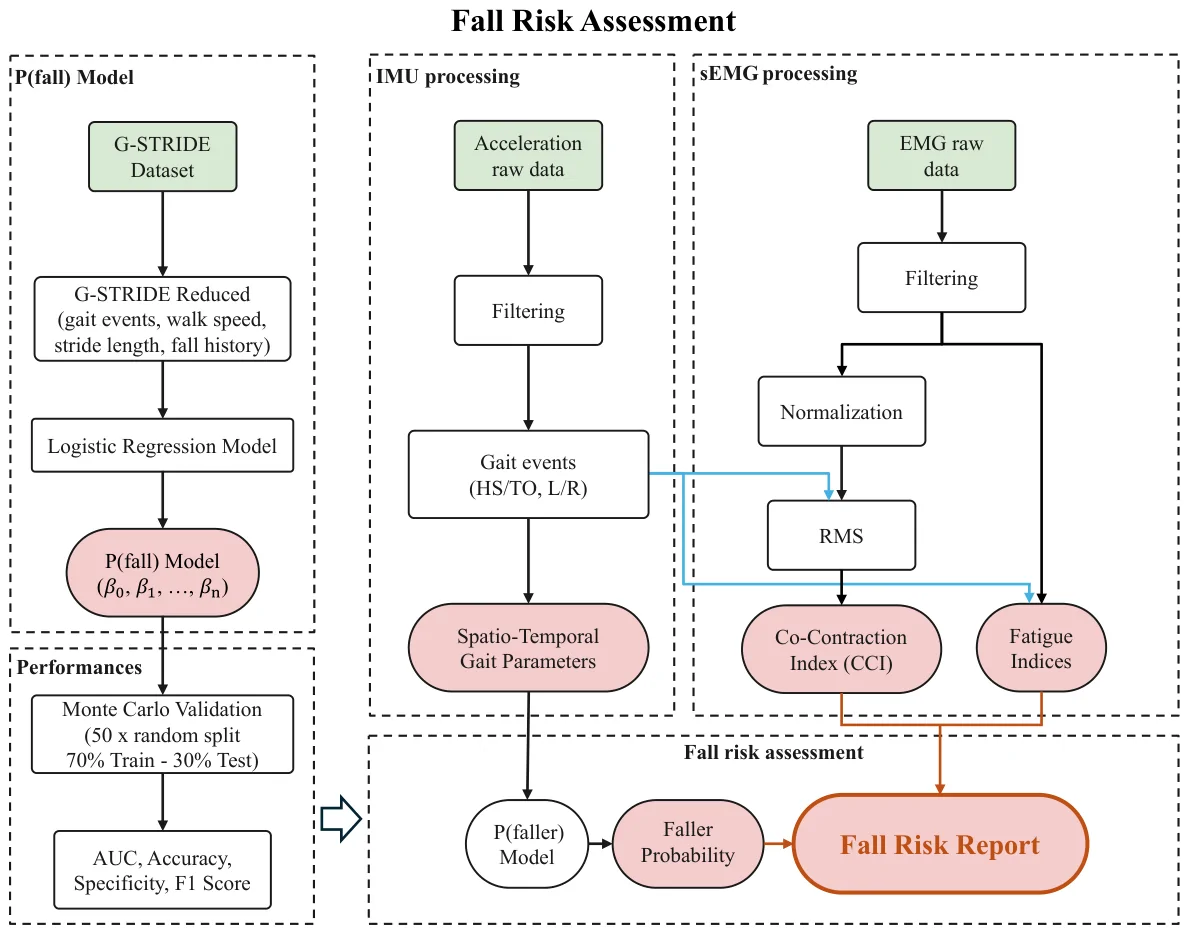
System workflow for fall risk assessment.

In this section, the methodological steps implemented for the development of the “Fall Risk Assessment” tool are presented. They include data collection and extraction of fall-risk-related parameters from kinematic and neuromuscular signals, with the definition of a model estimating the probability of belonging to the faller (vs. non-faller) group. The entire workflow is shown in Figure 2.

### 3.1. Data Collection

The database used for the Fall Risk Assessment is divided into two parts. The first one is a selection of IMU data and clinical data that come from an open-source database, described in Section 3.1.1. The second one is a new set of data collected with the MyoETA system, as described in Section 3.1.2.

#### 3.1.1. G-STRIDE Database

In order to obtain a model estimating the probability of belonging to the faller (vs. non-faller) group from spatio-temporal gait parameters, we used a publicly available dataset [45] originally described by Álvarez et al. [43] in an observational, multicenter case-control study on fall risk in older adults. The dataset comprises n=163 subjects (86 fallers, 77 non-fallers) and contains demographic data, clinical outcomes, together with gait kinematics measured with a foot-worn IMU during walking tests. From this database, we used the self-reported “Falls during the last year” field as the binary outcome label, and the IMU-derived kinematic parameters, in particular gait cycle time variability, swing percentage, cadence, walking speed, and stride length, as the predictive features of our model.

#### 3.1.2. Experimental Tests for Fall Risk Assessment

In order to test the developed model, a specific experimental phase was conducted in the ERGOS laboratory of the University of Naples Federico II. It involved a healthy male participant (age: 39; height: 166 cm; weight 62 kg). The characteristics of the MyoETA participant relative to the G-STRIDE reference population are reported in Table 1.

**Figure 3:**
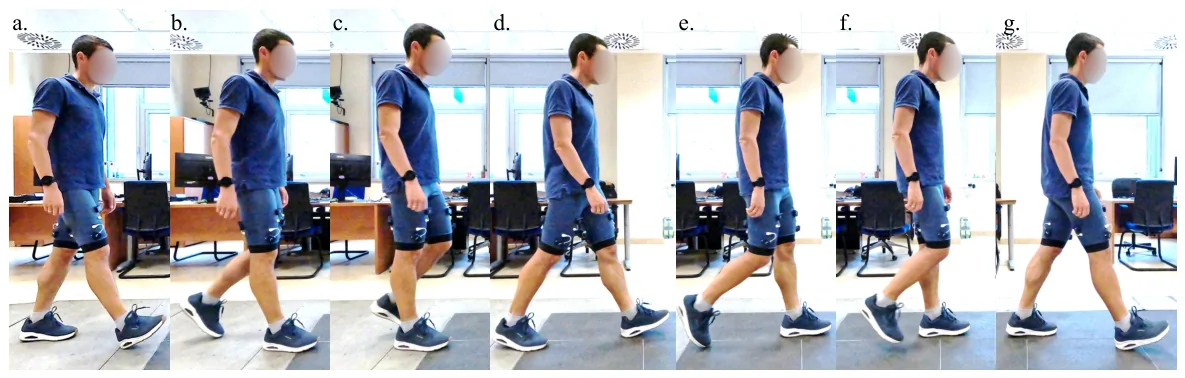
Sequence illustrating a complete gait cycle recorded during the 10 mWT, with the subject wearing the MyoETA sensorized shorts. In detail: (**a**) Right HS; (**b**) Left TO; (**c**) Right-leg midstance; (**d**) Left HS; (**e**) Right TO; (**f**) Left-leg midstance; (**g**) Right HS, marking the end of the gait cycle.

Prior to the study, his informed written consent was obtained, in accordance with the Ethical Committee Guidelines. Before starting the experimental task, the participant was required to wear the garment for at least 5 min. This settling period was introduced to allow stabilization of the electrode–skin interface, as previous studies have shown that dry and silicone-based electrodes achieve reduced impedance after an initial period of contact [41]. Proper wearing was ensured by aligning it with the anatomical landmarks, identified by the thermally pressed visual markers described in Section 2, in correspondence with the ASIS and PSIS. The task consisted of a level walking test: the participant was asked to stand still in an upright position for 5 s, then he was asked to perform a 10 mWT [7] as shown in Figure 3. It allows us to collect sEMG and IMU raw data.

Prior to this test, a preliminary mechanical characterization of the garment was performed: electrode-site stability and contact pressure were assessed during representative activities of daily living (treadmill walking at 6 km/h, sit-to-stand, stair climbing, and balance maintenance). Garment displacement relative to the anatomical landmarks was on the order of 0.5 mm. The contact pressure locally exerted at the electrode sites by the garment’s compressive structure was measured with a PicoPress pressure gauge (Microlab Elettronica, Padova, Italy). The average value was 10 mmHg, which is close to the optimal value reported to match the performance of Ag/AgCl electrodes for smart clothing with sEMG [46], and well below the 40 mmHg tolerability limit reported for compression garments [47].

### 3.2. Data Processing

Following data acquisition, raw signals were processed to extract IMU- and sEMG-derived metrics useful to assess fall risk. In detail, Section 3.2.1 reports the process to assess the main spatio-temporal gait parameters. These metrics are computed as key parameters to be used in a model estimating the probability of belonging to the faller (vs. non-faller) group. Section 3.2.2 reports the process to assess additional indicators related to muscle co-contraction and fatigue. These metrics are computed as additional key parameters associated with fall risk in the literature.

#### 3.2.1. Spatio-Temporal Gait Parameters

**Figure 4:**
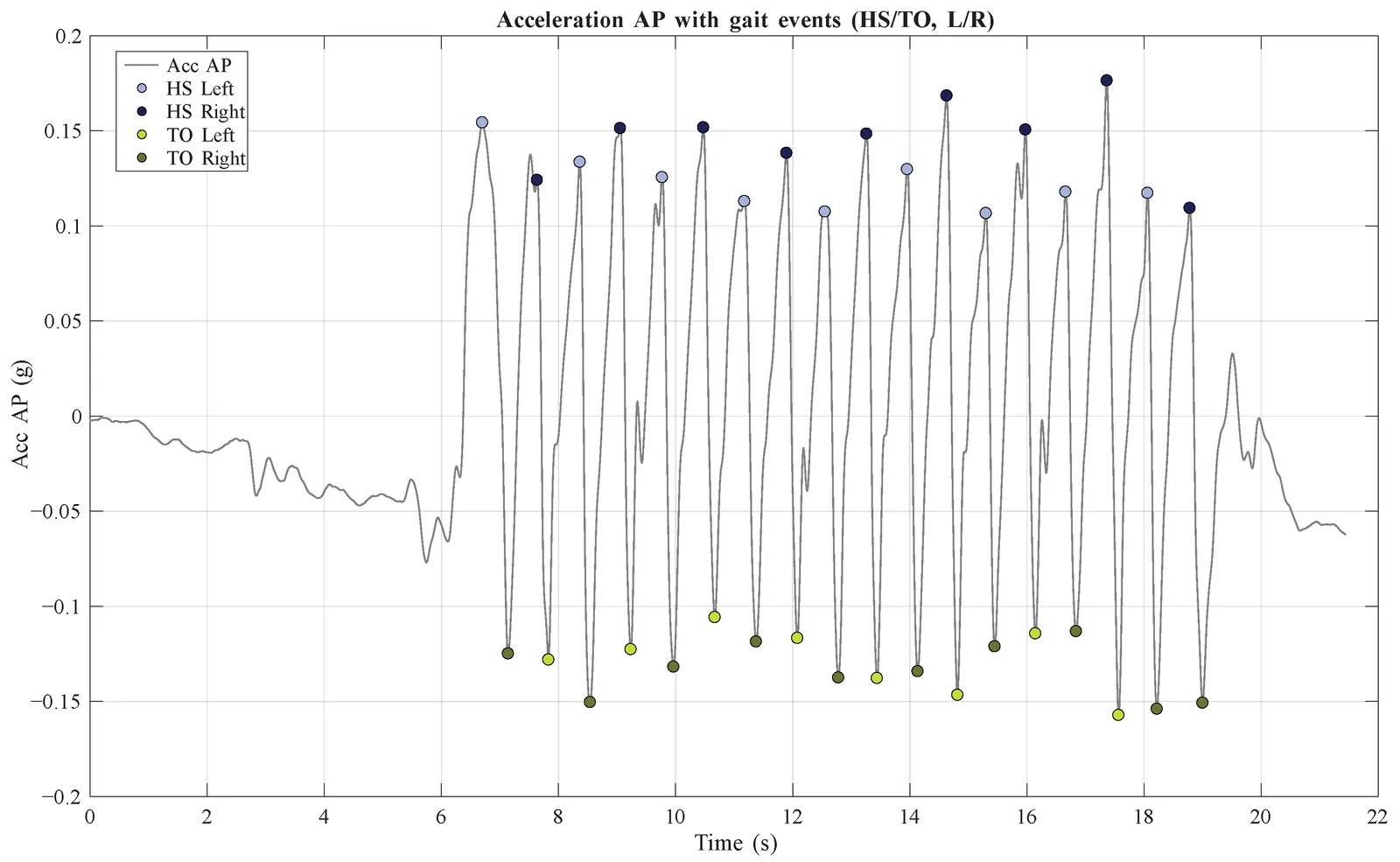
Filtered antero-posterior acceleration signal during 10 mWT with gait events detected in accordance with [16].

**Gait events** Prior to the computation of spatio-temporal gait parameters, gait events were detected from the IMU signal acquired at the L5 level. In order to obtain the typical antero-posterior (AP) acceleration pattern, characterized by two consecutive positive peaks followed by a negative peak (see Figure 4), raw data were filtered using a first-order Butterworth low-pass filter with a cut-off frequency of 2 Hz [16]. Then, regarding the gait events of interest for data segmentation, heel-strike (HS) of one limb side was identified in correspondence with the second positive peak, whereas toe-off (TO) of the opposite side was identified as the subsequent local minimum. Left and right gait events were discriminated using the acceleration curve along the medio-lateral (ML) axis. According to the inverted pendulum model for level walking, and assuming the sensor placement close to the CoM, accelerations to the left were associated with the right steps and vice versa.

**Temporal Gait Parameters** Once the HS and TO events were identified, the following temporal gait parameters were computed:

- *Gait Cycle (GC)* [s], the mean time interval between two consecutive HS of the same foot;
- $GC_{STD}$ [s], the standard deviation of the GC duration;
- *Swing* [%_GC_], the mean time interval between TO and the subsequent HS of the same leg, expressed as a percentage of the GC.
- *Cadence* [stride/min], obtained as the total number of strides per minute.

**Spatio-Temporal Gait Parameters** In addition, spatio-temporal gait parameters were assessed:

- *Walking speed* [m/s] was estimated using the model proposed by Byun et al. [48], developed for gait analysis using a single lumbar IMU.
- *Stride length* [m] was subsequently estimated by combining walking speed and cadence, according to the formula:
  (1) $$\mathrm{Stride}\;\mathrm{length}=\frac{\mathrm{Walking}\;\mathrm{speed}\times 60}{\mathrm{Cadence}}.$$

#### 3.2.2. Additional Neuromuscular Parameters

Raw sEMG signals acquired from the eight channels were first corrected for DC offset and filtered with a 50 Hz notch filter to remove powerline interference. A fourth-order Butterworth band-pass filter (10–450 Hz) was then applied, followed by full-wave rectification and a linear envelope, obtained through low-pass Butterworth filtering with a cut-off frequency of 3 Hz. Each enveloped signal was then normalized to the maximum muscular activity for that muscle recorded during the task.

Signal segmentation into gait cycles was performed using the gait events identified from the lumbar IMU and described in Section 3.2.1.

The muscle co-contraction index (CCI) quantifies the relative agonist-antagonist co-activation of the knee flexor and extensor muscles [49], and is defined as:

(2) $$\mathrm{CCI}=\frac{{\mathrm{RMS}}_{\mathrm{antag}.}}{{\mathrm{RMS}}_{\mathrm{antag}.}+{\mathrm{RMS}}_{\mathrm{ag}.}},$$

Since the RMS is itself an envelope estimator, it was computed directly on the band-pass filtered signal, prior to rectification and envelope extraction:

(3) $$\mathrm{RMS}\left(ph\right)=\sqrt{\frac{1}{N}\sum_{n=1}^{N}x_{ph}{\left[n\right]}^{2}},$$

where $x_{ph}\left[n\right]$ is the band-pass filtered sEMG signal within phase ph and *N* is the number of samples in that phase.

The RMS was computed separately over the stance and swing phases of the gait cycle, identified from the gait events described in Section 3.2.1. In each phase, ${\mathrm{RMS}}_{\mathrm{ag}.}$ and ${\mathrm{RMS}}_{\mathrm{antag}.}$ refer to the knee extensor and flexor muscles expected to contribute predominantly to, respectively, the extension movement sustained during stance and the flexion movement sustained during swing: during stance, ${\mathrm{RMS}}_{\mathrm{ag}.}$ is the sum of the RMS values of VL and RF (knee extensors) and ${\mathrm{RMS}}_{\mathrm{antag}.}$ is the sum of the RMS values of BF and ST (knee flexors); the roles are reversed during swing.

The median frequency (MDF) was computed for each detected stride and for each muscle, with right-leg strides used for the right-leg muscles and left-leg strides used for the left-leg muscles. Per-stride MDF values were grouped into consecutive blocks of eight strides, and the mean and standard deviation were computed.

In addition, an instantaneous-mean-amplitude (IMA) fatigue index was computed [50]. The segmented sEMG signal was band-pass filtered into a high-frequency sub-signal (HFSS) and a low-frequency sub-signal (LFSS); the IMA of each sub-signal was computed and the fatigue index obtained as their difference:

(4) $${\mathrm{Fatigue}}_{\mathrm{IMA}}={\mathrm{IMA}}_{\mathrm{LFSS},ph}-{\mathrm{IMA}}_{\mathrm{HFSS},ph},$$

(5) $$\begin{array}{cc} {\mathrm{IMA}}_{\mathrm{LFSS},ph} & =\sum_{n=0}^{N-1}\frac{|{\mathrm{LFSS}}_{ph}\left[n\right]|}{N},\;{\mathrm{IMA}}_{\mathrm{HFSS},ph}=\sum_{n=0}^{N-1}\frac{|{\mathrm{HFSS}}_{ph}\left[n\right]|}{N}, \end{array}$$

where ph is the analyzed phase and *N* the number of samples in the corresponding signal portion. This index was also computed for each detected stride and for each muscle, following the same left/right stride assignment described above. Muscle fatigue was considered present when ${\mathrm{Fatigue}}_{\mathrm{IMA}}$ reached or exceeded zero, following the criterion proposed by [50].

### 3.3. Data Analysis

Starting from the database described in Section 3.1.1, a probability model (i.e., P(faller) model) of belonging to the fallers (vs. non-fallers) group estimated from spatio-temporal gait parameters was obtained with the steps reported in Figure 2. In detail, among the models reported in [43], we selected the one based solely on IMU-derived parameters and from it we retained only the variables listed in Section 3.2.1 (with the exception of the GC). Given the lumbar placement of the IMU in the MyoETA system, this subset of parameters was selected as these were the variables that could be estimated with the greatest precision. Following the modeling approach of the original study, a binary logistic regression model was fitted to predict the probability of being classified as a faller, P(faller), from the five selected gait features. The model follows the standard logistic form

(6) $$P\left(\mathrm{faller}\right)=\frac{1}{1+e^{-\eta }}$$

where

(7) $$\eta =\beta_{0}+\beta_{1}\cdot GC_{STD}+\beta_{2}\cdot \mathrm{Swing}+\beta_{3}\cdot \mathrm{Cadence}+\beta_{4}\cdot \mathrm{Walking}\;\mathrm{Speed}+\beta_{5}\cdot \mathrm{Stride}\;\mathrm{Length}.$$

Model coefficients were estimated by maximum likelihood using the Logit implementation of the Python package statsmodels (v. 0.15.0) fitted on the full sample of 163 subjects. The binary outcome variable (faller/non-faller) was derived from the self-reported “Falls during the last year” field of the dataset.

Model performance was assessed using a repeated random subsampling (Monte Carlo cross-validation) scheme, following the validation methodology described in Álvarez et al. [43]. Specifically, the dataset was randomly split 50 times into a training set (70% of subjects, stratified by outcome) and a held-out test set (30% of subjects); for each split, the logistic regression model was refitted on the training set and evaluated on the corresponding test set, and the reported performance metrics are the mean across the 50 splits.

Model performance was assessed using the area under the ROC curve (AUC), accuracy, sensitivity, and specificity.

## 4. Fall Detection

**Figure 5:**
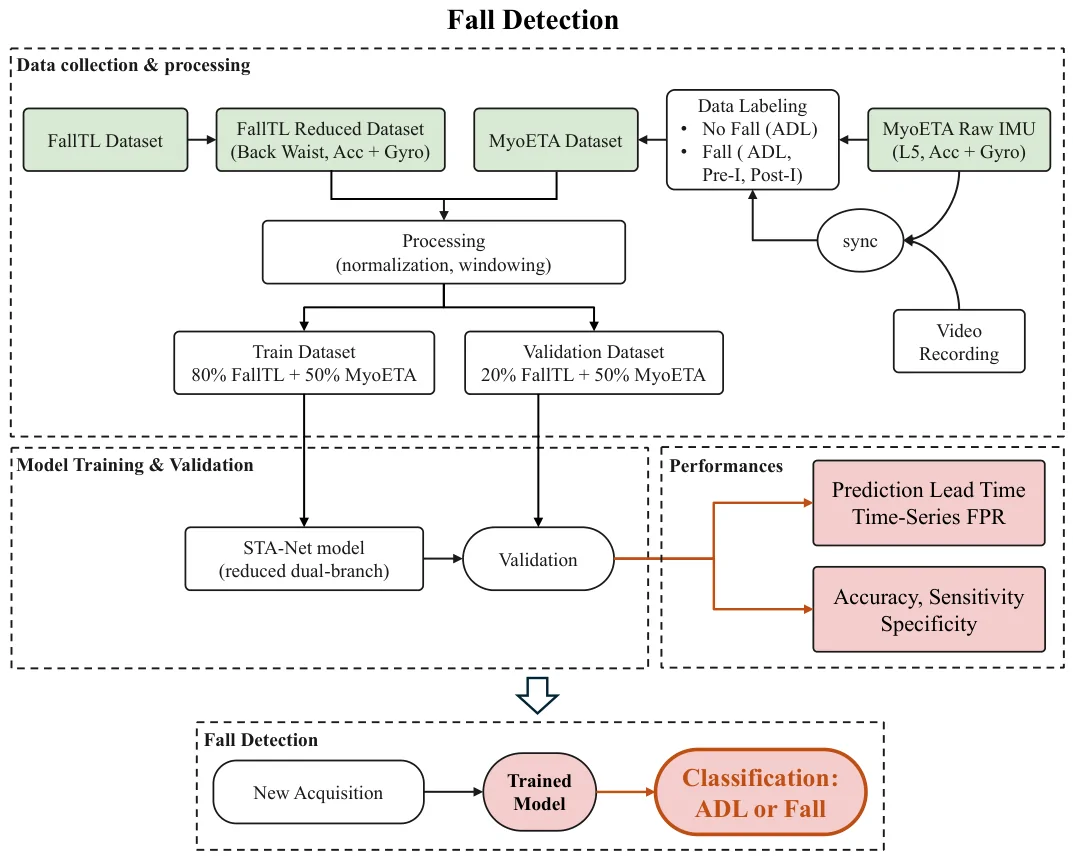
System workflow for fall detection.

In this section, the data collection, processing and analysis for defining fall detection is presented. We drew inspiration from the work of Cai et al. [44], who propose STA-Net (Spatio-Temporal Attention Network), a dual-branch neural network for both fall detection (FD) and pre-impact fall detection (PIFD). This study proved particularly relevant to our case, since in terms of accuracy, sensitivity, and specificity, its results indicate that: (1) trunk-mounted sensors (namely on the chest and waist) achieve the best detection performance, a region to which the lumbar (L5) placement of the MyoETA garment belongs; (2) although the configuration with accelerometer, gyroscope, and orientation (Euler) yields the highest PIFD performance, combining accelerometer and gyroscope signals already exceeds 80% accuracy for both PIFD and FD, consistent with the six-channel setup of MyoETA. The entire workflow is shown in Figure 5.

### 4.1. Data Collection

The database used for Fall Detection is divided into two parts. The first one is a selection of IMU data that come from an open-source database, described in Section 4.1.1. The second one is a new set of data collected with the MyoETA system, described in Section 4.1.2.

#### 4.1.1. FallTL Database

To implement a fall prediction pipeline, we implemented a reduced-size version of STA-Net and trained it on FallTL [51], a large-scale public dataset containing IMU data sampled at 200 Hz across eight body segments, acquired from 45 healthy young adults, comprising 34 fall activities and 12 ADLs.

In detail, the FallTL dataset was restricted to the back-waist sensor location, and only data from the triaxial accelerometer and gyroscope were considered.

#### 4.1.2. Experimental Tests for Fall Detection

The whole experimental phase, including ADLs and fall acquisitions, was conducted in a gymnasium. A single healthy subject (same participant in the fall-risk assessment experiment) wore MyoETA following the same recommendations reported in Section 3.1.2. The characteristics of the participant relative to the FallTL reference population are reported in Table 2.

**Figure 6:**
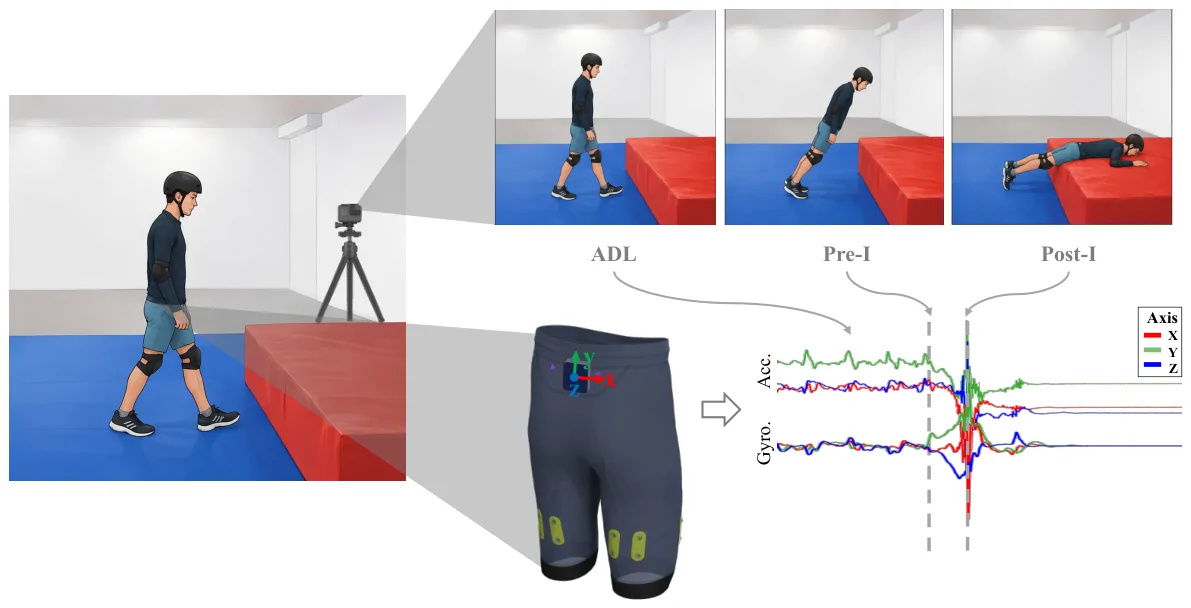
Experimental setup for the fall trials. Top: sequence representing a forward-direction fall during walking. Bottom: corresponding IMU data from the lumbar sensor unit (triaxial accelerometer and gyroscope) during the illustrated fall trial above, with dashed lines marking the onset of Pre-I and Post-I.

The participant was asked to perform all the ADLs and a subset of the fall scenarios included in the dataset described in [44]. To ensure his safety, falls were simulated using a high-jump landing mattress (200 × 200 × 40 cm), while wearing personal protective equipment such as a helmet, knee pads, and elbow pads. The fall timeline segmentation in fall sub-phases was obtained through synchronized IMU acquisitions and video recordings captured with a GoPro HERO10 Black (GoPro, Inc., San Mateo, CA, USA) set, with resolution equal to 2.7 K, frame rate equal to 240 fps and wide field of view. Alignment between the video and IMU streams was achieved using a finger flick applied to the sensor and recorded by the camera prior to each task execution. The described setup is shown in Figure 6.

Table 3 and Table 4 summarize the ADLs and falls recorded: 12 ADLs were tested; 12 falls tested were derived from 6 of the ADLs starting movements (standing, sitting, stand-up, sit-down, pick-up, and walking), each combined with two directions, forward (F) and lateral (L). Backward-direction falls were not tested, even if contained in the FallTL dataset, to reduce the risk of sensor damage on ground impact. Each ADL was performed three times and each fall twice, in a randomized order, for a total of 36 ADL and 24 fall trials (60 acquisitions overall), distributed across three separate sessions during the same day. In order to obtain the most natural fall patterns possible, the subject was given no instructions on how to perform them.

### 4.2. Data Processing

In order to exploit the FallTL dataset and the results reported for the neural network presented by Cai et al. [44], our own data needed to follow the same labeling scheme adopted in their work. Furthermore, while FallTL also distinguishes between recovery and non-recovery post-fall, this distinction was not considered here as the classifier developed in this work performs a binary classification (fall vs. non-fall). Therefore, for each of the 60 acquisitions collected with MyoETA (36 ADLs and 24 falls taken from the same subject, Section 4.1), raw accelerometer and gyroscope data were processed through the following pipeline before being passed to the classifier.

For the Video-IMU synchronization the finger flick on the sensing unit produced an impulsive acceleration peak on the IMU signal, while the corresponding instant of contact was simultaneously visible in the video recording. The video frame corresponding to this contact instant was manually identified by an operator through visual inspection using the open-source Kinovea software (version 2025.2, Joan Charmant and co.). This synchronization allowed the onset of the pre-impact phase (Pre-I), defined as the video frame in which the subject, while performing the ADL preceding the fall, began to exhibit a loss of balance and was therefore mapped onto the corresponding instant in the IMU time series. To identify the transition between Pre-I and Post-I, in accordance with [44], the impact instant corresponded to the moment of maximum sum magnitude vector (SMV), computed as the Euclidean norm of the three acceleration axes using a 100 ms moving average filter. Following the steps reported in Figure 5, data from the three-axis accelerometer and gyroscope were z-score normalized independently for each acquisition, using the mean and standard deviation computed per channel over the entire time series. Each normalized recording was segmented into fixed-length windows of 1 s (200 samples at 200 Hz), using a sliding-window approach. Whereas Cai et al. [44] adopted a 50% window overlap, a 95% overlap was adopted here, corresponding to a step size of 10 samples (50 ms), in order to obtain a prediction every 50 ms. Each window was labeled as “fall” if its time span overlapped the Pre-I phase, and as “non-fall” otherwise.

Table 5 summarizes the key hardware and pre-processing characteristics of the two IMU systems combined in this work: FallTL data were acquired with the JY901S (WIT Motion, Shenzhen, China) sensing module, while MyoETA was acquired with the BMI088 sensing module (see Section 2).

Once both datasets had the same labeling and pre-processing structure, training and validation sets were then defined via a stratified hold-out split for the reduced FallTL dataset. For the MyoETA subset, the split was defined as follows: since two acquisition sessions of fall trials were available (12 trials each, 24 total), one full session (12 trials) was assigned to the training set; for the ADLs, three acquisition sessions were available (12 trials each, 36 total), of which 18 trials (one full session and randomly selected 6 additional trials) were assigned to the training set. The remaining 12 falls and 18 ADLs trials were assigned to the validation set. The training set therefore combined 80% of FallTL with 50% of the MyoETA dataset, while the validation set comprised the remaining 20% of FallTL and 50% of MyoETA.

Per-channel normalization (mean and standard deviation) was computed exclusively on the training partition and subsequently applied to both training and validation, preventing information leakage from the validation set into the training pipeline.

### 4.3. Data Analysis

The fall detection module was trained in two configurations: one relying solely on the FallTL dataset, and one incorporating the MyoETA acquisitions.

The classifier presented in this work is based on a reduced-size version of STA-Net (GRU hidden size and convolutional width set to 16 vs. 64 of Cai et al. [44]); the reduction was adopted because it improved performance relative to the original dimensions, for which some overfitting was observed. The model was trained with a focal loss (α=0.25, γ=2.0) to counteract the class imbalance inherent to the pre-impact detection task, using a batch size of 2048, a learning rate of $1\times 10^{-3}$, and up to 150 epochs, with early stopping on validation loss (patience of 5 epochs). Training and inference were performed on a desktop computer (Intel Core i7-14650HX CPU, no GPU acceleration).

**Figure 7:**
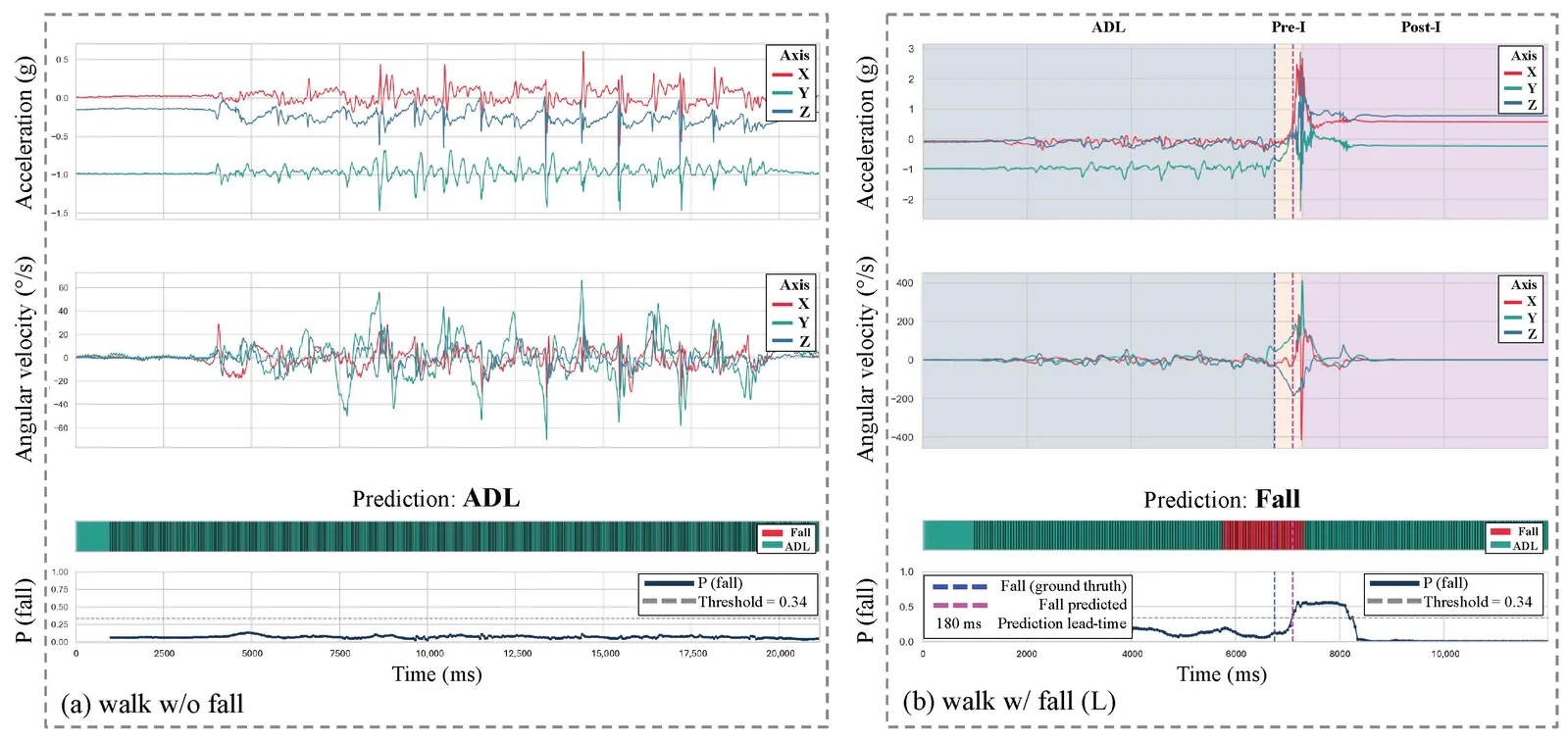
Model prediction for: (**a**) walking without falls, with P(fall) staying below the classification threshold; (**b**) walking with a lateral (L) fall, with ADL, Pre-I, and Post-I phases marked. Threshold crossing (magenta line) occurs 180 ms before ground impact and 350 ms after Pre-I onset (blue line).

Window-level outputs were expressed as a probability of fall, $P\left(\mathrm{fall}\right)=\mathrm{softmax}{\left(\mathrm{logits}\right)}_{1}$, i.e., the softmax output corresponding to the positive (fall) class, which was converted into a binary prediction by applying an operating threshold (0.34), selected on the validation set as the value maximizing the F1-score. A time series was labeled as containing a predicted fall if at least one of its overlapping windows exceeded the threshold; the prediction time used to compute the prediction lead time corresponds to the onset of the first such window. Two examples of activity classification are reported in Figure 7.

Model performance was assessed with the standard metrics of accuracy, sensitivity and specificity, alongside two metrics specific to time-series behavior: the prediction lead time, defined as the interval between the first predicted fall event and the onset of the post-impact phase (impact occurring just before Post-I), and the series-level false positive rate, defined as the fraction of ADL time series containing at least one predicted fall.

## 5. Results

In this section, the results obtained for the Fall Risk Assessment and Fall Detection tools are presented, following the methodological steps described in Section 3 and Section 4, respectively.

### 5.1. Fall Risk Assessment

The faller (vs. non-faller)-probability model was particularized starting from the dataset described in Section 3.1.1. Table 6 reports the estimated coefficients of the model, in accordance with Equation (7). Table 7 reports the assessed performance metrics according to the steps presented in Section 3.3. For reference, the original study reported comparable performance for their IMU-only model (Accuracy = 69%, Sensitivity = 72%, Specificity = 64%), despite relying on a considerably larger set of IMU-derived parameters (14 variables, including stance sub-phase durations, foot pitch angles, and 3D/2D path length, which are not estimable from an IMU placed on L5). Also, AUC can be considered in line with the reference that reported this value only for the best-performing model, which combined four conventional clinical variables with two IMU-derived kinematic variables (AUC = 77.6%).

The spatio-temporal parameters obtained from the 10 mWT presented in Section 3.2.1, and used to compute P(faller), are reported in Table 8. For this acquisition, the probability of belonging to the faller group, estimated in accordance with Equation (7) and the corresponding β coefficients (Table 6), resulted in a value of η = −1.30 ⇒ P(faller) = 0.214 (21.4%).

For the neuromuscular parameters, the CCI was computed for each detected stride, in accordance with the agonist/antagonist sub-division described in Section 3.2.2, separately for the stance and swing phases and for the left and right leg. Values are reported in Table 9 as mean ± SD across all strides within each phase/side combination. They were close across legs and gait phases, with low stride-to-stride variability. To assess muscle fatigue, MDF and IMA indexes were also computed for each muscle and each stride during the 10 mWT, following the left/right segmentation procedure described in Section 3.2.2, and subsequently averaged into a single mean ± SD value per muscle. Values are reported in Table 10. In addition, since IMA values ≥0 are indicative of muscle fatigue [50], the table reports the maximum IMA (IMA_max_) measured for each muscle. Median frequency and IMA fatigue indices remained stable across the trial, with IMA never reaching the ≥0 threshold associated with fatigue onset in any muscle (Table 10).

### 5.2. Fall Detection

Table 11 summarizes the two training configurations. The model trained solely on FallTL achieved strong window-level metrics on its own validation split (accuracy 96.4%, sensitivity 79.5%, specificity 98.1%, F1 = 76.5%), but transferred poorly to the MyoETA hardware at the time-series level: only 8 of 12 falls were detected, with highly variable prediction lead times (mean 140 ms, median 105 ms) and a series-level false-positive rate of 16.7% (3/18). Incorporating a weighted subset of MyoETA data (3×) into training slightly changed window-level metrics (accuracy 96.7%, sensitivity 78.0%, specificity 97.9%, F1 = 77.3%), and produced an improvement at the time-series level: all 12 falls were detected, prediction lead time increased (mean 390 ms, median 205 ms), and the false-positive rate decreased to 11.1% (2/18).

## 6. Discussion

The following subsections discuss the results obtained for each of the two developed tools.

### 6.1. Fall Risk Assessment (Part I)

The Fall Risk Assessment results presented in Section 5.1 should first be interpreted as a single-subject proof of concept, demonstrating that the proposed platform can reliably and repeatably acquire, transmit, and process data to extract spatio-temporal, kinematic, and neuromuscular parameters from an ADL such as walking.

The relatively low estimated probability—P(faller) = 21.4%—is consistent with expectations, given that the subject was a healthy, non-elderly adult with no reported fall history; no statistical claim can be drawn from a single acquisition. It is worth emphasizing that P(faller) estimates the probability that an individual gait pattern resembles that of the faller (vs. non-faller) group of the G-STRIDE reference study—defined from self-reported falls in the year preceding the walking test—rather than the probability that a fall will occur. In fact, gait parameters alone do not capture one individual’s lifestyle: a physically active person with relatively good function may belong to the faller group due to greater exposure, whereas an individual with impaired mobility may belong to the non-faller group partly because of restricted activity, even if their objective balance or mobility (e.g., low SPPB, prolonged TUG, or high concern about falling on the FES-I) would suggest otherwise.

The added value of the platform lies in the sEMG-derived neuromuscular parameters (Section 3.2.2), whose relevance to fall risk has been investigated in the literature, as discussed in Section 1 [23,24,25], and which are not available in G-STRIDE or in conventional IMU-only clinical assessments. Although these parameters do not modify the P(faller) probability estimate itself, they provide complementary information on the neuromuscular status of the patient. Whether this information translates into a more complete clinical picture, when combined with the gait-based probability estimate, is a question that remains to be tested in future work.

Regarding the neuromuscular fatigue indices, we are aware that fatigue assessment based on MDF is most informative when its trend is examined over an adequate stride window. The 10 mWT performed here, however, is a short task, and walking itself is not typically a fatiguing exercise for the proximal thigh muscles, particularly in a young, healthy adult. When examining the elderly population, however, the effects are different: Zhang et al. [52], for instance, found a significant decrease in EMG median frequency of the quadriceps femoris, hamstrings, and fibularis longus in a cohort of 18 older adults (63.4 ± 4.1 years) following a 60 min treadmill walking protocol performed at self-selected walking speed, with median frequency computed from 15 consecutive strides extracted at baseline and again at the 60th minute. The population and the number of strides available for a stable median-frequency estimate differ substantially from the present study, which limits direct comparison but also explains why fatigue signatures were not expected here. Consistently, the IMA fatigue index never reached values ≥0 for any muscle during the trial (Table 10), in line with the expectation that a 10 m walk is not a fatiguing task for a healthy subject.

Regarding the co-contraction index, we acknowledge that some of the monitored muscles are bi-articular and contribute, to some extent, to both the stance and swing phases rather than acting as pure agonists or antagonists; for this reason, muscles were assigned to the agonist or antagonist role based on their predominant contribution in each phase, as described in Section 3.2.2. Within this framework, the similarity of CCI values across the left and right leg and across gait phases (Table 9) is an indicator of good bilateral symmetry in muscle activation, while the low standard deviation observed indicates limited stride-to-stride variability in co-contraction, both consistent with a stable, physiological neuromuscular activation pattern expected from our subject. The neurophysiological mechanisms discussed in Section 1 offer some context for these indices. Age- and pathology-related declines in neural drive and automatic motor control would be expected to alter co-contraction and fatigue patterns during gait, so the stable, physiological values observed here likely reflect the subject’s young and healthy status rather than indicating low fall risk per se. Whether these indices shift in the expected direction in older adults, or in individuals with the pathological conditions discussed earlier, is a question that future work on a larger cohort should address.

### 6.2. Fall Detection (Part II)

The first configuration tested corresponded to using the STA-Net architecture and dataset from Cai et al. [44] essentially as proposed, which was evaluated directly against our own MyoETA acquisitions. Recognition performance in this configuration was not fully satisfactory: in some cases the fall was flagged well before the physiologically meaningful interval between Pre-I onset and impact, while in others it was flagged only after impact (down to −1025 ms among lateral falls), producing high variability in the resulting lead time despite a positive overall average; in addition, the failure to detect 4 of the 12 falls concentrated among lateral falls, which accounted for 3 of the 4 misses. This motivated incorporating part of our own data into the training set, with a higher weight (3×) assigned to the MyoETA windows relative to FallTL. This produced a clear improvement in performance on our own hardware.

It is worth clarifying why two different sets of metrics are reported in Table 11. The window-level accuracy, sensitivity, specificity, and F1-score are computed over the full validation split (FallTL + MyoETA) to allow a performance comparison in line with the reference study. However, what is genuinely informative for our purpose are the model performances on our data. This is why the time-series-level metrics were computed exclusively on the MyoETA validation subset. Among the hardware settings compared in Table 5, the accelerometer range represents the largest difference between the two systems (±3 g for MyoETA vs. ±16 g for FallTL’s JY901S). However, characterizing the 4/12 MyoETA validation falls missed by the FallTL-only model shows that missed falls exhibit lower peak accelerations and shorter pre-impact phases than detected ones, with three of the four occurring in the lateral direction; notably, walking was the only movement type for which both trials (forward and lateral) went undetected. This pattern suggests that the direction and kinematic profile of the fall—rather than the reduced accelerometer range—may be a more likely contributing factor, potentially reflecting differences in how the fall itself was physically performed. Cai et al. [44] do not specify how falls were executed in their protocol, which limits our ability to assess whether execution differences between the two studies play a role. Sensor placement could be an additional reason: a lower position (lumbar L5 in MyoETA vs. chest/waist in Cai et al. [44]) could itself result in lower peak accelerations during a fall, potentially contributing to the generally lower magnitudes observed in our data compared to FallTL. Another open variable relates to differences in how the sensor is affixed to the body. Testing falls under matched conditions and comparing performance to isolate these factors is a direction we plan to pursue in future work.

A terminological clarification is also necessary: throughout this analysis, a window is labeled as “fall” when it overlaps the Pre-I phase, not the impact event itself. This is intentional and central to the aim of this work: the goal is not to detect that a fall has occurred, but to recognize the pre-fall instability pattern before ground impact, so that a protective countermeasure can be triggered in time. The prediction lead time is, in our view, the most clinically relevant of these metrics, since it defines the time margin available for a protective intervention to be triggered before ground impact. Previous studies have reported lead times ranging from 40 to 750 ms [53]; as a point of reference, Shi et al. [54] proposed an airbag-based protection system with an inflation time of approximately 130 ms, already below the mean lead time obtained here (390 ms; median 205 ms) after incorporating MyoETA data, suggesting that the current system already provides a workable margin for this type of countermeasure, at least on average. However, triggering such a countermeasure in practice also requires the model to run fast enough on the target wearable hardware. Using the desktop setup described in Section 4.3, the reduced STA-Net (69,147 parameters, 0.26 MB) takes on average 10.1 ms per prediction—well within the reported lead time. This is a reduction relative to the original architecture [44] (206,618 parameters), but it was only tested on desktop hardware, and this alone does not show that the model is light enough to run on a microcontroller inside the garment. Table 12 compares our model against two recent studies that jointly consider detection performance and computational constraints for wearable deployment: TinyCNN [55], a quantized convolutional network evaluated on the KFall [56] and SisFall [57] datasets and reduced to 546 parameters to run in 37 ms on a real microcontroller (Arduino Nano BLE 33 Sense); and TinyFallNet [58], a lightweight pre-impact fall detection model trained on the young-subject KFall dataset [56] and evaluated on elderly data from the extended KFall ADL set [59] and the FARSEEING real-world fall repository [60], which achieved strong detection performance (see below) but was likewise not tested on embedded hardware in its original study.

Table 13 compares detection performance with the same two studies. Our window-level sensitivity (78.0%) is well below TinyCNN’s (99.8%/98.3%), most likely because our task is harder by design—we label only the pre-impact phase as a fall, excluding the impact itself, where the clearest signal changes occur—and because our training data (one MyoETA subject plus FallTL) is far smaller than the 32–38 subjects used in KFall/SisFall. Per-trial, our model detected all 12 falls—up from 8/12 with FallTL alone, a gain that may partly reflect subject-specific adaptation given the 3× weighting applied to the MyoETA subset during training—which is nonetheless in line with TinyFallNet’s 86.7% on elderly subjects. Our per-trial specificity (88.9%, 16/18), however, remains lower than TinyFallNet’s (96.8–98.0%); with only 18 ADL trials, a single false positive already changes this percentage by 5.6 points.

Future work will focus on expanding data collection of falls and ADLs to a larger cohort, and to use part of it to perform appropriate model fine-tuning, rather than the weighted loss-merging strategy adopted in the present work. Beyond increasing the model’s capacity to perform PIFD, the aim will be to further improve the prediction lead time, particularly for harder cases such as lateral falls or falls during walking, which contributed negatively to both the missed detections and the most negative lead times observed with the FallTL model only.

Finally, Table 14 summarizes the fall-related information that can currently be derived from the sEMG-only, IMU-only, and combined configurations of the MyoETA platform, including the Fall Risk Assessment tool. As already noted, the P(faller) model relies on IMU-derived gait parameters, while sEMG-derived neuromuscular indices are currently reported alongside it to further support kinematic information (inside the Fall Risk report). Fall Detection, instead, relies on lumbar-IMU data alone, and sEMG’s potential contribution remains unexplored.

Given the relevance of the neuromuscular component in fall dynamics, particularly in the context of PIFD, a specific opportunity lies in the pre-impact phase itself (in order to develop an Improved PIFD). Prior work has shown that sEMG can support real-time recognition of loss-of-balance events, with detection times compatible with triggering a protective response [61]. Notably, two of the muscles used for this purpose in that study (rectus femoris and biceps femoris) are already monitored bilaterally by MyoETA for the CCI computation (Section 3.2.2), suggesting these same channels could be explored as additional predictors for the Fall Detection module. This direction is further supported by prior work on distal lower-limb muscles, namely the tibialis anterior and gastrocnemius, already employed for pre-impact fall detection [34,35].

## 7. Limitations

This study is subject to several limitations that should be addressed in future works. First, both the Fall Risk Assessment and the Fall Detection experiments were conducted on a single, healthy participant. Consequently, no certainty of generalizability can be drawn from either module.

For the Fall Risk Assessment module, the outcome variable used to train the model reflects self-reported fall occurrence. Developing a model capable of directly estimating fall risk will require validation on a larger cohort, including older participants with a documented fall history and standardized clinical assessments of balance and mobility (e.g., SPPB, TUG, FES-I), together with follow-up to track future fall incidence.

For the Fall Detection module, the improvement observed in performance after incorporating MyoETA data (Section 5.2) may reflect subject-, device-, or environment-specific adaptation rather than a generalizable gain, an interpretation reinforced by the 3× weighting applied to MyoETA samples during training. In addition, both the training and the validation MyoETA subsets originate from the same participant described in Section 4.1.2.

Second, all falls used to train and evaluate the Fall Detection module, both in FallTL and in the MyoETA acquisitions, are simulated and performed by young, healthy adults; this inevitably introduces a bias with respect to the target population. Among the ADLs recorded (Table 3), the shuffling gait was intended, following the FallTL protocol, to approximate gait alterations often seen in older adults, but performed by a young, healthy volunteer, it remains at best an approximation; the same limitation extends to the simulated falls themselves, since the neurophysiological mechanisms discussed in Section 1 would be expected to affect not only the gait preceding a fall but the fall event itself. Collecting real, unplanned fall data from elderly subjects, while preserving their safety, remains an open and unresolved challenge in the field; one direction consistent with the literature is to collect only ADL data from the target population and use transfer learning to adapt the model without exposing participants to fall risk, as demonstrated by Maray et al. [62] for fall-detection personalization.

## 8. Conclusions

In this work, we presented a wearable platform to address both fall risk assessment and fall detection based on a sensorized garment integrating IMU and sEMG sensors. Part I extracts spatio-temporal gait and neuromuscular parameters from a 10 mWT to estimate the probability of belonging to the faller (vs. non-faller) group via a logistic regression model trained on a reduced set of variables from the G-STRIDE dataset, achieving cross-validated performance (AUC = 77.8%, accuracy = 68.7%) in line with the reference study; results obtained on a single healthy subject demonstrated the feasibility of the acquisition and processing pipeline as a proof of concept. Part II addresses the feasibility of continuously monitoring and distinguishing ADLs from the onset of a fall, using a reduced Spatio-Temporal Attention Network (STA-Net) trained on FallTL—restricted to the back-waist sensor location and to accelerometer and gyroscope data, matching MyoETA sensor configuration—and refined with data acquired directly with the platform from the same single participant introduced in Part I. This addition improved within-subject performance on acquisitions with the platform (12/12 falls detected, average prediction lead time of 390 ms), providing a workable margin for triggering protective intervention. Taken together, these results support the proposed system as a unified platform integrating objective fall-risk screening with real-time pre-impact detection, addressing a gap in current wearable solutions targeting the elderly and fall prevention, where the two functions are typically treated separately. Future works will focus on validating both the faller-classification and fall-detection modules on larger cohorts, including data from unseen participants and elderly individuals with a documented history of falls. Additionally, the integration of IMU- and sEMG-derived information into both the fall-risk assessment and fall-detection modules will be explored, as the current models rely solely on IMU data.

## Figures and Tables

**Table 1:** Demographic and anthropometric characteristics of the G-STRIDE reference population compared with the MyoETA participant.

Characteristic	G-STRIDE Cohort (n=163)	MyoETA Participant (n=1)
Sex, female	72.4% (n=118)	Male
Age [years]	82.63 ± 6.01 (range 70–98)	39
Height [m]	1.56 ± 0.10	1.66
Weight [kg]	64.28 ± 13.11	62
Fallers/non-fallers	86/77	—

**Table 2:** Demographic and anthropometric characteristics of the FallTL reference population [44], compared with the MyoETA participant.

Characteristic	FallTL Cohort (n=45)	MyoETA Participant (n=1)
Sex, female	13.3% (n=6)	Male
Age [years]	22.24 ± 1.48	39
Height [cm]	176.07 ± 7.42	166
Weight [kg]	74.79 ± 20.89	62

**Table 3:** Activities of daily living (ADL) recorded with MyoETA. Each ADL was performed 3 times, for a total of 36 trials.

#	ADL	#	ADL
1	Standing	7	Jogging
2	Sitting	8	Lie-down
3	Lying	9	Get-up
4	Side lying	10	Pick-up
5	Walking	11	Sit-down
6	Shuffle	12	Stand-up

**Table 4:** Fall trials recorded with MyoETA. Each starting ADL was combined with two fall directions, forward and lateral, each performed twice, for a total of 24 trials.

Starting ADL	Direction	
	Forward	Lateral
Standing	✓	✓
Sitting	✓	✓
Walking	✓	✓
Pick-up	✓	✓
Sit-down	✓	✓
Stand-up	✓	✓

**Table 5:** Comparison of the IMU measurement systems used in FallTL [44] and MyoETA.

Feature	FallTL—JY901S (WIT Motion)	MyoETA—BMI088 (Bosch Sensortec)
Interface	I2C	I2C or SPI (configurable)
Sampling frequency	200 Hz	200 Hz
Acc. range	±16 g	±3 g
Acc. resolution	0.5 mg/LSB	0.09 mg/LSB
Gyro. range	±2000°/s	±2000°/s
Gyro. resolution	0.061°/s per LSB	0.061°/s per LSB
Calibration	Not reported	Factory-calibrated

**Table 6:** Logistic regression coefficients (full-sample fit, n=163).

Coeff.	Estimate
$\beta_{0}$	3.8545
$\beta_{1}$	4.6476
$\beta_{2}$	−0.0469
$\beta_{3}$	0.0029
$\beta_{4}$	−3.1498
$\beta_{5}$	−0.4368

**Table 7:** Cross-validated performances of the logistic regression model to estimate probability of belonging to the faller (vs. non-faller) group.

Metric	Value (Mean)
AUC	77.8%
Accuracy	68.7%
Sensitivity	73.9%
Specificity	62.9%

**Table 8:** Spatiotemporal gait parameters during a 10 m walk test in a healthy subject.

Metric	Value (Mean)
$GC_{STD}$ [s]	0.068
Swing [%_GC_]	36.42
Cadence [stride/min]	42.66
Walking speed [m/s]	1.03
Stride length [m]	1.47

**Table 9:** Co-contraction index (mean ± SD) during the stance and swing phases of the gait cycle, for the left and right leg.

Gait Phase	Left Leg	Right Leg
Stance	0.63±0.04	0.59±0.06
Swing	0.40±0.03	0.34±0.09

**Table 10:** Neuromuscular parameters for each monitored muscle during the 10 mWT: median frequency (MDF) and IMA fatigue index, reported as mean ± SD, with the maximum IMA value attained during the trial.

Muscle	MDF [Hz] (Mean ± SD)	IMA (Mean ± SD)	IMA_max_
RF-L	117.85±12.03	−0.476±0.172	−0.12
BF-L	123.92±43.58	−0.058±0.016	−0.02
VL-L	181.54±18.65	−0.074±0.005	−0.07
ST-L	089.17±14.68	−0.116±0.069	−0.05
RF-R	182.53±25.67	−0.071±0.003	−0.07
BF-R	087.00±22.47	−0.122±0.047	−0.06
VL-R	146.55±51.02	−0.083±0.027	−0.06
ST-R	114.72±20.93	−0.084±0.035	−0.06

**Table 11:** Time-series–level performance: results on the MyoETA garment test set (12 falls, 18 ADLs; single subject), with and without incorporating the weighted subject-specific series into the training set.

	FallTL Only	FallTL + MyoETA (3×)
Window-level metrics (validation split)		
Accuracy	96.4%	96.7%
Sensitivity	79.5%	78.0%
Specificity	98.1%	97.9%
F1 score	76.5%	77.3%
Time-series metrics (MyoETA validation data only)		
Falls detected	8/12	12/12
Mean prediction lead time (ms)	140	390
Median prediction lead time (ms)	105	205
Series-level FPR	16.7% (3/18)	11.1% (2/18)

**Table 12:** Computational complexity and deployment characteristics of the reduced STA-Net compared with lightweight wearable pre-impact fall-detection models from the literature.

Model	Params	Size	MACs/win.	Latency	Hardware
STA-Net (reduced)	69,147	0.26 MB	75.1 M	10.1 ms	Desktop CPU
STA-Net (orig. scale)	206,618	0.79 MB	139.9 M	—	—
TinyCNN [55]	546	—	—	37 ms	Arduino (MCU)
TinyFallNet [58]	—	0.70 MB	—	—	Not embedded

**Table 13:** Comparison of detection performance with lightweight wearable fall-detection studies from the literature.

Model	Task	Population	Level	Sens.	Spec.	Lead Time
STA-Net (+MyoETA)	PIFD	1 subj., 39y	Window	78.0%	97.9%	—
			Series	100% (12/12)	88.9% (16/18)	390 ms
STA-Net (FallTL only)	PIFD	1 subj., 39y	Window	79.5%	98.1%	—
			Series	66.7% (8/12)	83.3% (15/18)	140 ms
TinyFallNet [58]	PIFD	Young	Series	99.3%	96.8%	539 ms
		Elderly	Series	86.7% (13/15)	98.0% (627/640)	478 ms
TinyCNN [55]	FD	KFall, quant.	Window	99.8%	99.2%	—
		SisFall, quant.	Window	98.3%	98.5%	—

**Table 14:** Fall-related information currently derived from sEMG, IMU, or their combination (i.e., IMU+sEMG) on the MyoETA platform. X indicates an available feature; O indicates a not available feature.

	Output	sEMG Only	IMU Only	IMU+sEMG
Fall Risk Assessment	Spatio-temporal gait parameters	O	X	X
	Faller-classification, P(faller)	O	X	X
	Muscle co-contraction (CCI)	X	O	X
	Muscle fatigue (MDF, IMA)	X	O	X
	Fall Risk report	O	O	X
Fall Detection	Pre-impact fall detection (PIFD)	O	X	X
	Early neuromuscular instability signs	X	O	X
	Improved PIFD	O	O	X

## Data Availability

The data presented in this study are available on request from the corresponding author. The data are not publicly available due to privacy policy.

## Abbreviations

The following abbreviations are used in this manuscript:

10 mWT	10 m Walking Test
ADL	Activities of Daily Living
AP	Antero-Posterior
ASIS	Anterior Superior Iliac Spine
AUC	Area Under the Curve
BBS	Berg Balance Scale
BF	Biceps Femoris
BLE	Bluetooth Low Energy
CCI	Co-Contraction Index
CoM	Center of Mass
FD	Fall Detection
FES-I	Falls Efficacy Scale-International
FPR	False Positive Rate
GC	Gait Cycle
GRU	Gated Recurrent Unit
HFSS	High-Frequency Sub-Signal
HS	Heel-Strike
IMA	Instantaneous-Mean-Amplitude
IMU	Inertial Measurement Unit
LFSS	Low-Frequency Sub-Signal
MDF	Median Frequency
ML	Medio-Lateral
PIFD	Pre-Impact Fall Detection
PSIS	Posterior Superior Iliac Spine
RF	Rectus Femoris
RMS	Root Mean Square
ROC	Receiver Operating Characteristic
sEMG	surface ElectroMyoGraphy
SENIAM	Surface Electromyography for the Non-Invasive Assessment of Muscles
SMV	Sum Magnitude Vector
SPPB	Short Physical Performance Battery
ST	Semitendinosus
STA-Net	Spatio-Temporal Attention Network
TO	Toe-Off
TUG	Timed Up and Go
VL	Vastus Lateralis
WHO	World Health Organization
